# Differential Tissue Fatty Acids Profiling between Colorectal Cancer Patients with and without Synchronous Metastasis

**DOI:** 10.3390/ijms19040962

**Published:** 2018-03-23

**Authors:** Maria Notarnicola, Dionigi Lorusso, Valeria Tutino, Valentina De Nunzio, Giampiero De Leonardis, Gisella Marangelli, Vito Guerra, Nicola Veronese, Maria Gabriella Caruso, Gianluigi Giannelli

**Affiliations:** 1 Laboratory of Nutritional Biochemistry, National Institute of Gastroenterology-Research Hospital, Via Turi, 27, 70013 Castellana Grotte, Bari, Italy; valeria.tutino@irccsdebellis.it (V.T.); valentinadx@hotmail.it (V.D.N.); giampierodl@gmail.com (G.D.L.); ilmannato@gmail.com (N.V.); gabriella.caruso@irccsdebellis.it (M.G.C.); 2 Division of Surgery, National Institute of Gastroenterology-Research Hospital, Via Turi, 27, 70013 Castellana Grotte, Bari, Italy; dionigi.lorusso@irccsdebellis.it (D.L.); gisella.marangelli@live.it (G.M.); 3 Clinical Trial Unit, National Institute of Gastroenterology-Research Hospital, Via Turi, 27, 70013 Castellana Grotte, Bari, Italy; vito.guerra@irccsdebellis.it; 4 Scientific Direction, National Institute of Gastroenterology-Research Hospital“S. de Bellis”, Via Turi, 27, 70013 Castellana Grotte, Bari, Italy; gianluigi.giannelli@irccsdebellis.it

**Keywords:** colon cancer, metastasis, lipidomic analysis, fatty acids

## Abstract

The early detection of colorectal cancer and determination of its metastatic potential are important factors to set up more efficacious therapeutic strategies. In the present study, we hypothesize that fatty acids analysis in colorectal cancer patients can discriminate between metastatic and non-metastatic patients. Fifty-one consecutive patients with histologically proven colorectal cancer were enrolled in the study and the presence of synchronous metastasis was detected in 25 of these 51 patients. Fatty acid profile analysis in red blood cell membranes was not able to discriminate the metastatic colorectal cancer patients from those without metastasis. However, significant differences in the tumor tissue fatty acid profile were found in metastatic cancer patients when compared to patients without metastasis. Metastatic patients showed significantly lower percentages of Eicosapentaenoic acid (EPA) and higher levels of γ-linolenic acid (GLA), a *n*-3- and *n*-6-Polyunsaturated fatty acid (PUFA), respectively. Our findings, suggesting that membrane lipid rearrangement could influence the cellular function and make the cell more prone to metastasis, offer the opportunity to develop nutritional strategies that may be helpful in the prevention and treatment of colorectal cancer.

## 1. Introduction

Colorectal cancer (CRC) localized only at the primary site is generally curable by surgical resection, but if the tumor has spread to distant sites, the patient five-year survival rate declines quickly [1]. Thus, the early detection of CRC and determination of its metastatic potential are important factors to set up more efficacious therapeutic strategies. Several molecular biomarkers have been associated with CRC progression and the development of metastases [2,3].

Lipid metabolism is known to influence tumor growth and, in general, dyslipidemia has been associated with an increased risk for CRC [4,5]. Previously, we demonstrated that high serum levels of low-density lipoprotein cholesterol (LDL-C) and triglycerides are associated with a higher presence of metastases in CRC patients [6]. Other studies have documented the role of LDL in intestinal tumorigenicity, showing that LDL induces intestinal inflammation via activation of reactive oxygen species (ROS) and the mitogen-activated protein kinase (MAPK) pathway [3,7,8].

In addition, an altered expression of the fatty acids pattern has been demonstrated to be a crucial event in colorectal carcinogenesis and progression [9,10,11]. Fatty acid synthase (FAS) activity levels, as well as mRNA expression, are upregulated in colorectal cancer tissues [12]. Recently, in an in vivo study, we showed that a possible molecular mechanism by which omega-3-polyunsaturated fatty acids(*n*-3-PUFAs) and olive oil in the diet were able to reduce cell proliferation and increase apoptosis in the intestinal polyps of Apc Min/+ mice was the reduction of lipogenic enzymes activity and gene expression, such as FAS [13].

FAS is a key enzyme in the fatty acids biosynthesis pathway; fatty acids and their polyunsaturated derivatives have a structural role in cell membranes, influencing their fluidity and physiological functions. Thus, the study of fatty acids composition of the cell membrane can be considered an appropriate biomarker for investigating the relations between the lipid metabolism and specific diseases [14]. In our preliminary study, we demonstrated the presence of an altered fatty acid profile in patients with CRC compared to subjects without malignant diseases; a reduction of total *n*-3-PUFAs levels and consequently a higher *n*-6-PUFAs/*n*-3-PUFAsratio was detected in cancer patients compared to control subjects [15].

Moreover, the nature of lipids in the diet has been demonstrated to profoundly modify cell membrane structure and function, suggesting that dietary lipids influence the protein transduction processes involved in cell proliferation, apoptosis and differentiation [16].

Considering the current evidence, the study of metabolic and lipidomic profiling can be considered a powerful tool to identify useful biomarkers to predict cancer progression and the development of metastases.

Several experimental and clinical studies have shown that metabolic profiles closely reflect the cellular environment where the tumor develops [17,18,19]. In addition, these studies have shown that cancer cell analyses, using metabolomic techniques, are able to establish the tumor metastatic potential with an important effect on metastasis control [20].

In the present study, we hypothesize that lipidomic analysis in CRC patients can discriminate between metastatic and non-metastatic patients. Therefore, the aim of this study was to perform a comparative analysis of the fatty acid profile in red blood cells membranes, as well as in tissue samples of CRC subjects with and without synchronous metastases.

## 2. Results

Table 1 shows clinical characteristics of the CRC patients enrolled in the study. No significant differences were found regarding age, sex, and body mass index (BMI) between the two CRC patient groups (data not shown). The presence of synchronous metastasis, evaluated using computed tomography (CT), was detected in 25 of 51 patients; metastases were in the liver for 10 patients, in visceral lymph nodes for 13 patients and 2 patients had bone and lung metastases, respectively.

**Figure 1:**
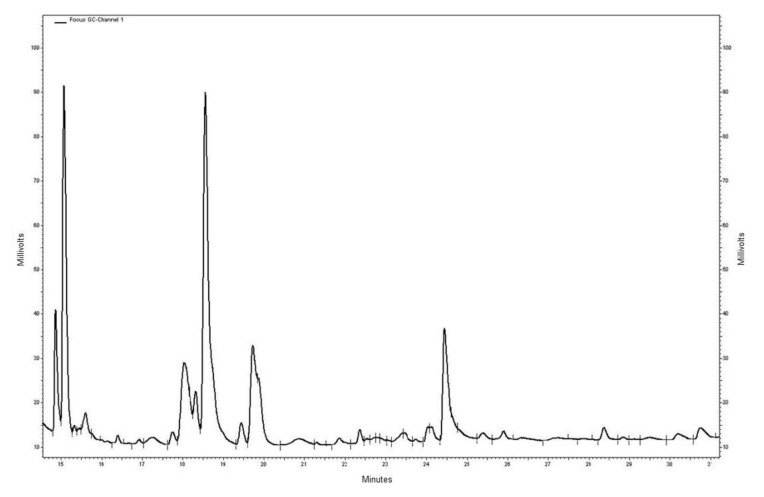
Representative chromatographic tissue fatty acids profile; the peaks corresponding to GLA and EPA were detected at the retention times of 20.853 min and 25.913 min, respectively.

Figure 1 shows a representative fatty acids profile detected using the gas chromatography method. Fatty acid profile analysis in red blood cell membranes is not able to discriminate metastatic CRC patients from those without metastasis (Table 2). However, significant differences in the tissue fatty acid profile were found in metastatic CRC patients when compared to patients without metastasis (Table 3). Metastatic CRC patients showed significantly lower percentages of Eicosapentaenoic acid (EPA) and higher levels of γ-linolenic acid (GLA), a *n*-3- and *n*-6-polyunsaturated fatty acid (PUFA), respectively. Consequently, the *n*-6-PUFAs/*n*-3-PUFAs ratio observed in CRC patients with metastasis was significantly higher compared to CRC patients without metastasis.

The differences in fatty acids levels observed in tumor tissue were not found in corresponding normal mucosa, even if a slight increase in the SFA, *n*-6-PUFA/*n*-3-PUFA ratio, and GLA levels, as well as a decrease in EPA levels, were detected in metastatic CRC patients compared to other patients group. This finding might be explained by the hypothesis that the presence of metastasis is the result of metabolic alterations that mainly occurred in the tumor cell.

No significant difference between the groups was observed in terms of the fatty acids profile as regard total SFAs, MUFAs, PUFAs, and the saturation index (SI) value, defined as the ratio between stearic/oleic acid and known to be an indicator of membrane fluidity.

## 3. Discussion

Starting from our previous observations demonstrating a significant increase of serum lipid levels in CRC patients with metastasis [6], in this study we confirm that not only do modifications in lipid metabolism occur in colorectal cancer, but that the presence of synchronous metastases was associated with a different tissue fatty acids profile compared to the profile detected in colorectal tissue of non-metastatic patients. The differences were more widely visible in tumor tissue than in corresponding normal mucosa, suggesting an important role of tumor-induced impairment in the formation of the cell membranes lipid structure.

Several points of evidence show that tumor growth and progression are affected by the complex interactions between cancer cells and the surrounding microenvironment [21,22,23,24]. Previously, we have demonstrated that tumor-associated factors, such as cytokines, growth factors or cellular receptors can influence lipid metabolism in peritumoral adipose tissue [24]. Moreover, in an animal model of colon carcinogenesis, we demonstrated that cancer cell metabolism is affected by dietary natural compounds, which are able to control and to improve the environmental conditions where tumors develop [25].

Metastatic CRC patients showed significantly lower percentage of EPA, an *n*-3-PUFA, known for its efficacy in regulating the lipid metabolism and for its anti-proliferative effects in vitro and in vivo [13,25,26]. EPA has been demonstrated to exert anti-proliferative effects through the regulation of HMGCoA reductase gene expression and of lipogenic enzymes belonging to the cholesterol biosynthetic pathway [27].

The deficit of EPA detected in the tissue of metastatic CRC patients is in agreement with studies showing the role of *n*-3-PUFAs as modulators of cell membrane lipid composition. The type of fatty acid incorporated into the phospholipids of the cell membrane is indicative of its architecture and its physiology. The membrane lipids rearrangement, influencing the cellular function and responsiveness to internal and external signals, could make the cell more suitable to metastasize [28].

In this study, another cell membrane metabolite which contributes to discriminating between metastatic and non-metastatic CRC patients is GLA, a *n*-6-PUFA derived from its essential fatty acid C18 precursor Linoleic Acid (LA). Potentially an increase of GLA in cell membrane enriches tissues with arachidonic acid (AA) because of their metabolic link. AA has been demonstrated to be a potent pro-inflammatory and pro-thrombotic signaling regulator with a detrimental effect on human health [29]. Dietary *n*-6-PUFAs seem to enhance cell invasion and metastasis, altering the environment within host target sites [28,29,30]. Collectively, these data suggest a relationship between high levels of GLA and the presence of metastasis in our patients.

A limitation of the present study is the inability to discriminate the presence of metastasis through the analysis of fatty acids profile in circulating erythrocytes, since risk factors of metastasis—including depth of tumor invasion, histological grading, and lymphovascular invasion—were not adjusted for comparison. Probably, in the future, the study of the fatty acid profile in red blood cells membranes of a larger number of CRC patients could help us to identify characteristic metabolic profiles of metastasis.

However, an innovative aspect of our study is represented that, here, we demonstrate the ability of tissue fatty acids analysis to identify lipid metabolism alterations and show thatthese are associated with CRC and with synchronous metastasis. Moreover, our study offers an opportunity to improve the development of nutritional strategies, in particular those aimed at maintaining the membrane lipid balance at optimal values, which can be used both in the prevention and the treatment of neoplastic diseases, such as CRC.

## 4. Materials and Methods

### 4.1. Patients 

Fifty-one consecutive patients (29 males and 22 females, mean age 68.3 ± 13.3), with histologically proven colorectal cancer hospitalized at the surgery division at our institute, were enrolled in the study. All patients were invited to give a blood sample prior to surgery for in vitro isolation of erythrocytes; in addition, at surgery, colorectal mucosa and cancer tissue were obtained from each of them and the specimens were taken and stored at −80 °C until assayed.

Informed consent was obtained from each patient and the study was approved by the Ethics Committee of IRCCS “S. de Bellis”, Castellana Grotte (Bari, Italy, number code: 32/CE/DE BELLIS, 27 October 2016).

### 4.2. In Vitro Isolation of Erythrocytes

Blood samples collected in tubes containing ethylenediamine-tetraacetic acid (K-EDTA) anticoagulant were quickly layered on a Ficoll–Paque solution and centrifuged at 400× *g* for 40 min at 20 °C. The lymphocytes and plasma were then removed and the erythrocytes were recovered from the bottom layer and washed with four volumes of phosphate-buffered saline. Isolated red blood cells were stored at −80 °C until they were assayed.

### 4.3. Fatty Acids Extraction and Preparation ofFatty Acid Methyl Esters from Erythrocytes and Tissue Samples 

We used the modified method of Moilanen [31], that is itself a modification of the method described by Folch [32,33]. Fatty acids extraction and preparation of fatty acid methyl esters(FAME) from red blood cells (RBC) and tissue samples were carried out as previously described [16,34]. Briefly, total lipids from phospholipids of RBC membranes were extracted by adding 0.9 mL of an acidified salt solution (H_2_SO_4_ 2 × 10^−4^ M, NaCl 0.1%). For fatty acids extraction from tissues, about 20 mg wet tissues were homogenized with 0.8 mL of ice cold 0.9% NaCl. All samples received 5.0 mL of chloroform:methanol (2:1, *v*/*v*) (Sigma-Aldrich, Milan, Italy) and the samples were mixed thoroughly and centrifuged at 1000× *g* for 10 min. The lower layer, containing fatty acids, was removed with care, replaced in a new tube and dried by a centrifugal evaporator (Bio-Rad, Milan, Italy). The FAME were obtained by adding toluene and BF_3_·MeOH 14% (Sigma-Aldrich, Milan, Italy) and incubating for 2 h at 80 °C. After the addition of toluene and 5% aqueous sodium chloride solution, the samples were centrifuged at 470× *g* for 10 min. Fatty acid methyl esters contained in the upper layer of the tubes, were collected and transferred into a vial and analyzed.

### 4.4. Gas Chromatography and Fatty Acids Quantification

Fatty acids quantification was performed by using a gas chromatography equipment with auto-sampler, a split/splitless injector, FID detector and a hydrogen gas generator (Thermo Fisher Scientific, Milan, Italy). Separation of FAME was carried out as previously described [16,34]. A BPX 70 capillary column SGE Analytical Science, P/N SGE054623, 60 m × 0.25 mm ID, BPX70 0.25UM (SGE Europe Ltd., Milton Keynes, UK) was used and the amount injected was 1 μL in splitless mode (split flow 50 mL·min^−1^, splitlesstime 1 min). Quantification of fatty acid methyl esters was performed using a mixture of standards (Supelco 37-Component FAME Mix, Sigma-Aldrich, Milan, Italy).

### 4.5. Statistical Analysis

All data were expressed as Mean ± SD. Wilcoxon rank-sum (Mann–Whitney) was performed to estimate differences between groups. A probabilistic type I error of ≤0.05 was considered as statistically significant. Statistical analysis was performed with StataCorp. 2007. Stata Statistical Software: Release 12 (StataCorp LP, College Station, TX, USA).

## Figures and Tables

**Table 1:** Clinical and histopathological features of colorectal cancer patients

Patients (*n* = 51)	
Age (mean ± SD)	68.3 ± 13.3
Sex	
Male	29
Female	22
BMI (kg/m^2^) ^a^	22.2 ± 4.03
Tumor Side ^b^	
Right	16
Left	35
Tumor Stage ^c^	
Stage I	7
Stage II	17
Stage III	16
Stage IV	11
Histological Grading	
Well-differentiated (G1)	2
Moderately-differentiated (G2)	27
Poorly-differentiated (G3)	22
Metastases	25
No metastases	26

^a^ Body mass index; ^b^ Right side: hepatic flexure, cecum and ascending colon; Left side: descending colon, sigmoid, and rectum; ^c^ Clinical staging performed using UICC System.

**Table 2:** Mean percentage of fatty acid composition of red blood cell membranes in CRC patients (51 cases).

	CRC Patients		
Fatty acids	No Metastases	Metastases	*p*-Value °
SFA	55.02 ± 6.2	53.7 ± 8.3	0.92
MUFA	22.02 ± 3.8	21.11 ± 4.1	0.81
PUFAs	23.94 ± 5.6	25.1 ± 3.9	0.75
n6/n3	4.5 ± 1.6	4.6 ± 1.2	0.52
Stearic acid	21.5 ± 4.6	22.3 ± 6.71	0.85
Oleic acid	12.7 ± 2.3	12.9 ± 3.2	0.95
SI *	1.9 ± 0.5	2.2 ± 1.4	0.88
GLA	0.03 ± 0.1	0.09 ± 0.2	0.25
EPA	0.44 ± 0.58	0.53 ± 0.3	0.70
DHA	2.76 ± 1.5	2.8 ± 1.4	0.96

* Saturation index (stearic acid/oleic acid ratio); ° Wilcoxon rank-sum (Mann–Whitney) test; All values are expressed as mean ± standard deviation.

**Table 3:** Mean percentage of fatty acid composition of tissue cell membranes in CRC patients (51 cases).

	Normal Mucosa			Tumor Tissue		
Fatty acids	No Metastases	Metastases	*p*-Value °	No Metastases	Metastases	*p*-Value °
SFAs	42.09 ± 5.1	47.54 ± 7.8	0.62	49.97 ± 4.2	48.53 ± 3.9	0.84
MUFAs	40.54 ± 3.5	38.22 ± 4.0	0.73	32.20 ± 2.7	33.71 ± 4.3	0.90
PUFAs	17.37 ± 2.0	15.99 ± 1.9	0.60	17.83 ± 3.1	16.7 ± 0.8	0.77
*n*-6/*n*-3	8.85 ± 5.82	15.10 ± 16.2	0.40	6.2 ± 4.5	15.2 ± 9.2	*0.04*
Stearic acid	15.2 ± 6.8	18.8 ± 7.8	0.22	18.56 ± 7.5	19.6 ± 6.7	0.71
Oleic acid	28.2 ± 8.4	35.5 ± 6.6	0.30	18.6 ± 4.5	21.3 ± 3.9	0.62
SI *	0.95 ± 1.5	0.72 ± 1.42	0.51	0.98 ± 0.49	1.5 ± 0.7	0.86
GLA	0.16 ± 0.6	0.26 ± 0.8	0.83	0.09 ± 0.11	0.34 ± 0.2	*0.05*
EPA	0.47 ± 0.6	0.36 ± 0.7	0.32	0.99 ± 0.54	0.46 ± 0.54	*0.002*
DHA	0.4 ± 0.35	0.2 ± 0.18	0.30	0.69 ± 0.48	0.54 ± 0.6	0.24

* Saturation index (stearic acid/oleic acid ratio); ° Wilcoxon rank-sum (Mann–Whitney) test; All values are expressed as mean ± standard deviation.
